# Oxidative balance in follicular fluid of ART patients of advanced maternal age and blastocyst formation

**DOI:** 10.5935/1518-0557.20200012

**Published:** 2020

**Authors:** Ricardo Pella, Silvia Suárez-Cunza, Patricia Orihuela, Francisco Escudero, Ygor Pérez, Mario García, Ingrid Zorrilla, Paola Berrío, Sergio Romero

**Affiliations:** 1 Centro de Fertilidad y Reproducción Asistida CEFRA, Lima, Peru; 2 Instituto de Bioquímica y Nutrición, Universidad Nacional Mayor de San Marcos, Lima, Peru; 3 Laboratorio de Biología Reproductiva y Preservación de la Fertilidad, Universidad Peruana Cayetano Heredia, Lima, Peru

**Keywords:** IVM, oxidative stress, oocyte, chromosome alignment

## Abstract

**Objective:**

To evaluate the follicular fluid oxidative balance of infertile patients of advanced-maternal-age and the correlation between oxidative imbalance in the follicular fluid and the embryological outcomes.

**Methods:**

Follicular fluid (FF) from infertile patients of advanced-maternal-age undergoing ART treatments were collected and frozen in cryovials at -86°C until further use, and analyzed at the Biochemistry and Nutrition Institute of San Marcos University. As controls, we used FF from oocyte donors. The FF was then assayed for oxidative balance by ABTS, FRAP and TBARS assays. In order to establish the correlation between oxidative balance and embryo quality, we correlated the number of usable blastocysts (freshly transferred or frozen blastocysts) with the results from ABTS, FRAP and TBARS.

**Results:**

Follicular fluid from patients of Advanced-maternal-age (AMA group) significantly differed from the values found in the control group; the ABTS value was higher and the FRAP value was lower, in comparison to the FF from oocyte donors (control group). The lipid peroxidation was not different between those two groups. Furthermore, there was no significant correlation among the results of the assays, or when correlated with the proportion of usable blastocysts.

**Conclusion:**

Ovarian oxidative balance seems to be critical for oocyte quality in advanced-maternal-age patients; however, we still need more studies on oxidative stress indicators, on a larger set of patients.

## INTRODUCTION

Follicular Fluid (FF) composition is complex, from the bloodstream it flows into the capillaries of the ovarian cortex and the components are secreted by the granulosa cells lining the interior of the ovarian follicle (Hennet & Combelles, 2012; Rodgers & Irving-Rodgers, 2010). It provides the oocyte with an environment to grow and enables its nuclear and cytoplasmic maturation, which is essential for its fertilization capacity and early embryonic development. In addition, the follicular fluid plays an important role in various reproductive processes associated with follicular growth, ovulation, oocyte nutrition, sperm capacitation and fertilization, as reported by Basuino & Silveira (2016).

This fluid is mainly composed of proteins and hormones, including follicle-stimulating hormone (FSH), luteinizing hormone (LH), growth hormone (GH), human chorionic gonadotropin (hCG), progesterone and estradiol; cytokines, anticoagulant enzymes, electrolytes, reactive oxygen species (ROS); enzymatic and non-enzymatic anti-oxidants, such as vitamin E, catalase and melatonin; growth factors such as epidermal growth factor (EGF), EGF-like factors and transforming growth factor alpha (TGF-α); metabolites such as amino acids and lipids that accumulate inside the oocyte, promoting its differentiation; and fatty acids (de Resende *et al.*, 2010; Hennet & Combelles, 2012; Hsieh *et al.*, 2009; Shaaker *et al.*, 2012).

An appropriate balance of the follicular fluid components would be necessary for the oocyte to properly mature; this includes an adequate oxidative balance. Granulosa cells, and macrophages (and cytokines) that produce reactive oxygen species (ROS), which in turn interact with lipids, proteins and nucleic acids causing peroxidative damage (Agarwal *et al.*, 2012; 2014; Sugino, 2005). Under normal conditions the so-called scavengers function as natural anti-oxidants, maintaining an oxidative balance that makes folliculogenesis and oogenesis, leading to the production of a healthy oocyte (Tamura *et al.*, 2008), eventually capable of generating an embryo, and pregnancy.

However, there are conditions in which oocyte quality is affected, giving rise to a poor-quality embryo (making it difficult to conceive), or leading to miscarriages due to chromosomal aberrations. Examples of such conditions are pathological conditions (such as endometriosis) or physical conditions (such as advanced-maternal-age) (Borges *et al.*, 2015; Harb *et al.*, 2013; Sauer, 2015).

It has been hypothesized that the poor-quality oocyte found in women with endometriosis and with advanced-maternal-age may be due to an ovarian oxidative imbalance (Agarwal *et al.*, 2012). There are several ways (all-indirect) to establish the oxidative balance in fluids. Among the most used are TBARS (Thiobarbituric Acid Reactive Substances), FRAP (Ferric Reducing Antioxidant Power) and ABTS [2, 2’-azino-bis (3-ethylbenzothiazoline-6-sulphonic acid)]. TBARS was developed more than 40 years ago and used to detect lipid peroxidation. This procedure measures the malondialdehyde (MDA), a byproduct of the degradation of hydroperoxides generated by lipid oxidation. FRAP was originally developed by Benzie & Strain (1996) to measure the reducing (antioxidant) power of plasma. The ATBS assay was developed by Miller *et al*. (1993). It is based on the ability of an antioxidant to stabilize a colored cation radical (ABTS•+), which is obtained by ABTS oxidation by metmyoglobin and hydrogen peroxide. The results are expressed as Trolox Equivalent Antioxidant Capacity (Londoño, 2012).

A deficient oocyte maturation in terms of oxidative imbalance could produce alterations in the reproductive processes previously described, as well as the generation of poor-quality embryos, which can be evaluated during early *in-vitro* embryonic development until the blastocyst formation.

In the current study, we evaluated the correlation between oxidative imbalance in the follicular fluid and the embryological outcomes.

## MATERIALS AND METHODS

### Collection of follicular fluid

The study included ART patients of advanced-maternal-age. We took off the study those patients with additional conditions (i.e. hydrosalpinx, non-infectious diseases) or more than one diagnosis. Additionally, we used follicular fluid from donors of our oocyte donation program as controls (control group). Both groups of patients underwent controlled ovarian stimulation (COS) with gonadotropins, until at least 3 developing follicles reached 17mm. At that moment, we triggered ovulation with GnRH agonist. The patients (or donors) received a total gonadotropin dose of around 2000 IU.

The Control Group had 10 patients, and the advanced-maternal-age group had 22 patients. The Ethics committee of the Universidad Peruana Cayetano Heredia approved the informed consent (Project number: 64957).

We collected the follicular fluid (FF) from the first follicle during Ovum Pick-Up (OPU) from the patients undergoing ART treatments. The rationale for using FF from the first follicle is that this FF was less likely to be contaminated by blood cells and flushing medium. We assumed that this follicle’s characteristics represented the ovary and/or the patient. Lamb *et al*. (2010) reported a similar approach. Moreover, this sample was representative for the environment to which the oocytes were subjected within the ovary. Following collection, we spun the FF at 3000 RPM x 10min. The supernatant was collected, frozen in cryovials at -86°C, prior to further use.

### Oxidative balance determination in follicular fluid

We sent the follicular fluid (FF) samples to be analyzed at the Biochemistry and Nutrition Institute of the San Marcos University. The follicular fluid samples were assayed by the ABTS, FRAP and TBARS assays. While, both ABTS and FRAP assess the antioxidant capacity of the sample, TBARS assesses lipid peroxidation.

The ABTS test measures the total antioxidant capacity (TAC) of a sample and it is based on the ABTS^• +^ radical discoloration. The cationic radical ABTS • + is a chromophore that absorbs at a wavelength of 734 nm and is generated by an oxidation reaction of ABTS (2,2'-azino-bis- (3-ethylbenzthiazolin-6-ammonium sulfonate) with potassium persulfate. Absorbance was evaluated at a wavelength of 734 nm after seven minutes. As a reference sample, we used a solution of the radical ABTS • + with the solvent of the sample, and the initial absorbance was 0.7±0.02. We report the results as TEAC values (trolox equivalent antioxidant capacity).

The FRAP test measures the ability of a sample to reduce ferric iron (Fe + 3) to ferrous iron (Fe + 2). The ferric iron 20 mM was mixed with a solution of 2,4,6-tri (2-pyridyl) -striazine 10 mM prepared in HCl 40 mM, in 300 mM acetate buffer, pH 3.6. The FRAP values are obtained at 593 nm after an incubation period of ten minutes at room temperature. The reference curve was constructed using ascorbic acid as the primary standard. The activities of the samples were expressed as nmol Eq Ascorbic Acid/ml.

The TBARS test aims to determine the degree of lipid peroxidation (indicative of oxidative stress). We combined each sample with 10% trichloroacetic acid (TCA), and 0.67% TBA prepared in 0.25 N HCl. The mixture sample plus TCA was heated in boiling water for 15 minutes, then with TBA for 30 minutes and centrifuged for 10 minutes at 10,000g. The solution’s absorbance was 535 nm. We express the values as µmol MDA/mL, using the extinction coefficient 1.56 x 10^-5^ M^-1^cm.^-1^

### Correlation between oxidative balance and embryo quality

To establish the correlation between oxidative balance and embryo quality, we correlated the number of usable blastocysts (total number of embryos that develop until the blastocyst stage that had been either freshly transferred or frozen for further use) with the ABTS, FRAP and TBARS results. We evaluated the developing embryos on the Day 5, for developmental stage and quality, based on the degree of the blastocoel cavity expansion, the size and compaction of the inner cell mass and the homogeneity of the trophoblastic cells (Gardner *et al*., 2009). The FF and embryos derived from our oocyte donation program were considered as controls.

### Statistics

We compared the values obtained from the ABTS, FRAP and TBARS tests between the control group (FF from donors from our oocyte donation program) and the Advanced-Maternal-Age (AMA) group using the Mann-Whitney test. The relationship between the number of usable blastocysts (freshly transferred or frozen blastocysts) was correlated with the ABTS, FRAP and TBARS results, and analyzed by the Pearson correlation. A *p*-value below 0.05 was statistically significant. We performed the statistical analyses using the GraphPad Prism version 7 for windows, GraphPad Software, La Jolla California USA.

## RESULTS

### Oxidative balance in the follicular fluid

Antioxidant (ABTS and FRAP) capacity as well as lipid peroxidation (TBARS) of follicular fluid was performed as described in materials and methods.

**Figure 1:**
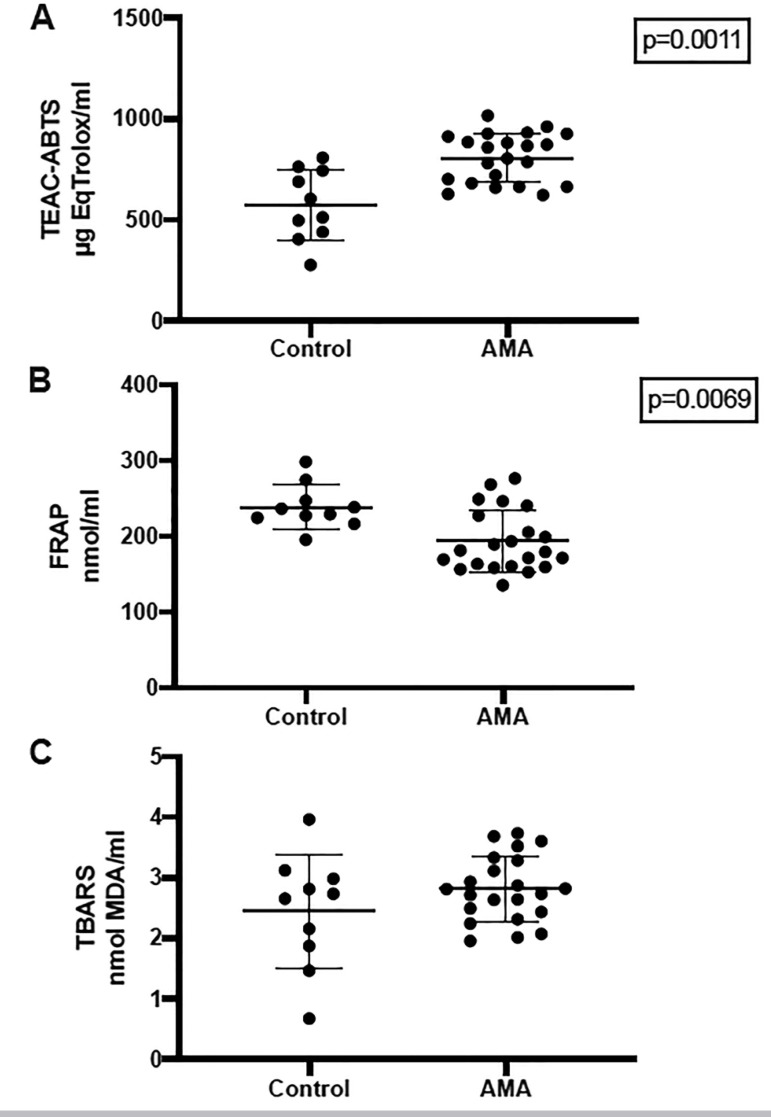
Scatter dot plot of ABTS (A), FRAP (B) and TBARS (C) values in both groups (Control and Advanced-maternal-age AMA patients) The Control group is represented by samples from donors from our oocyte donation program. The values are represented in specified units. Statistical significance is described within the graph.

According to the antioxidant capacity, the follicular fluid of advanced-maternal-age patients (AMA group) significantly differed from the values found in the control group (Figure 1 A & B). Remarkably, the total antioxidant capacity (measured by ABTS) was higher in the AMA group, and the FRAP value was lower, both compared to the control group. There were no differences in lipid peroxidation between the groups (as assessed by TBARS test) (Figure 1C).

**Figure 2:**
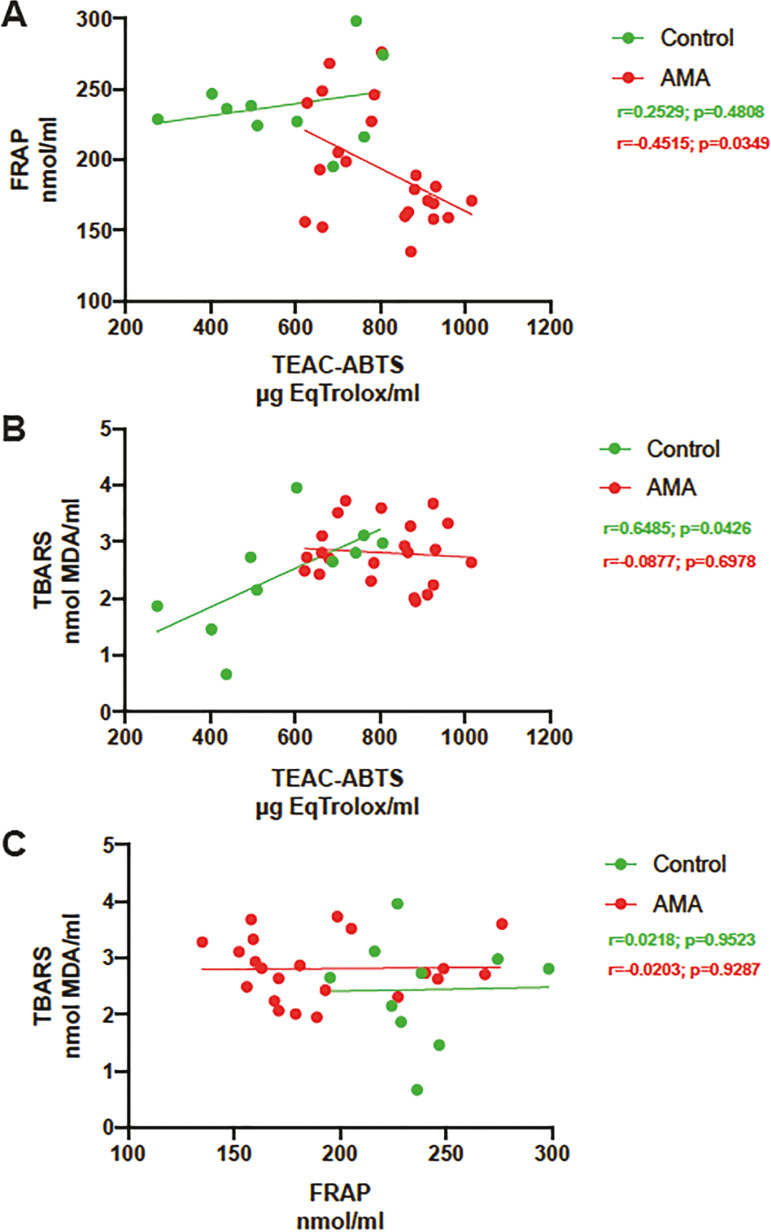
Correlation between ABTS, FRAP and TBARS values for both groups (Control and Advanced-maternal-age AMA patients) The Control group is represented by samples from donors from our oocyte donation program. The values are represented in the specified units. Statistical significance is described in the graph. A) ABTS vs FRAP; B) ABTS Vs TBARS; C) FRAP Vs TBARS

We evaluated the correlation between the results of the three assays; however, there was no significant correlation for any of the assays in either of the groups (Control or AMA) (Figure 2).

### Correlation between oxidative balance and embryo quality

**Figure 3:**
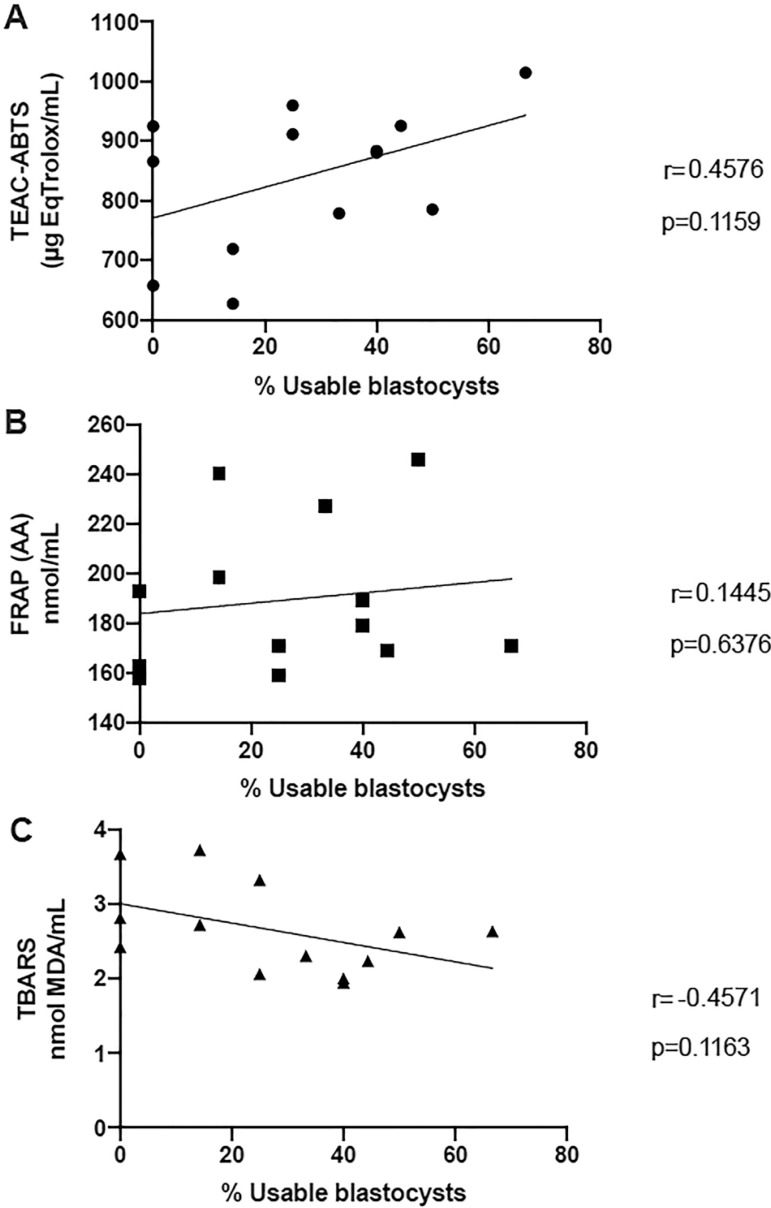
Correlation between the ABTS, FRAP and TBARS values of patients with Advanced-maternal-age and percentage of usable blastocysts The values are represented in the specified units. Statistical significance is described in the graph. A) ABTS; B) FRAP; C) TBARS

In an attempt to evaluate the impact of the oxidative imbalance found in the follicular fluid of the patients in the AMA group (compared to oocyte donors), the correlation between the percentage of usable blastocysts (either freshly transferred or frozen for deferred embryo transfer) and ABTS, FRAP and TBARS results was investigated. However, there was no significant association (Figure 3).

## DISCUSSION

High levels of reactive oxygen species in the follicular fluid are normally produced during the ovulation process (Shkolnik *et al.*, 2011). These are produced by phagocytic macrophages, parenchymal and endothelial cells. This physiological situation is counter arrested by an antioxidant defense system, which prevents oxidative stress (ovarian redox homeostasis); however, the antioxidant response is usually variable depending on several factors (Aydogan Mathyk *et al.*, 2018).

In contrast, a moderate level of oxidative stress serves as an intracellular signaling that favors cell proliferation; nevertheless, increased levels are counterproductive. The oxidative stress level in the FF depends on various causes; therefore, results found in the literature are quite heterogeneous (Huang *et al.*, 2015).

ART results are affected by different factors; many of them have not yet been fully investigated. Some studies reported that a lower antioxidant capacity affects gamete development. For example, the production of oxidative agents (i.e. nitric oxide) is higher in infertile patients with endometriosis who cannot become pregnant (Goud *et al.*, 2014; Singh *et al.*, 2013). In addition, with advancing age, there is a decrease in the systemic antioxidant capacity (Agarwal *et al.*, 2012; Eichenlaub-Ritter *et al.*, 2011), which may impact the quality of eggs and embryos (Tarin *et al.*, 1998; Zhang *et al.*, 2006). Moreover, Superoxide dismutase is lower in young ART female patients, while catalase is lower in ART patients of advanced-maternal-age (Wdowiak & Wdowiak, 2015).

Although the FF from obese and non-pregnant ART patients affects oocyte developmental competence and embryo quality in a bovine model (Valckx *et al.*, 2015), oxidative stress is negatively associated with pregnancy outcomes when evaluated systemically (serum) rather than locally (FF) (Di Rosa *et al.*, 2016). Similarly, there is no link between ROS in FF and embryo quality in ART patients (Siristatidis *et al.*, 2016). Additionally, the results from oxidative stress in FF of infertile PCO patients seem to be contradictory (Yilmaz *et al.*, 2016). Nevertheless, studies demonstrating that FF from ART patients (i.e. diagnosed with endometriosis) have lower antioxidant capacity than in other infertile patients (i.e. tubal occlusion, PCOS) (Huang *et al.*, 2014), more studies should be carried out to investigate these patients further.

Our results demonstrate that there is an altered antioxidant capacity in our patients with advanced-maternal-age; however, both techniques (ABTS and FRAP) yielded opposite trends. Since both techniques rely on electron, transfer, this apparent contradictory result may be explained by the composition and stoichiometry of the antioxidant components present in the follicular fluid of both groups.

Moreover, in contrast to a report describing that oral administration of antioxidants lowers oxidative stress in the FF of advanced-maternal-age patients, by increasing the total antioxidant capacity in serum and FF (Luddi *et al.*, 2016); no differences were found among patients that received (or not) oral antioxidants in the present study (data not shown).

The fact that the TBARS test has not shown a significant difference in both groups may imply that age (a factor that boosts ROS production) does not affect the redox homeostasis of the follicular fluid, which is in line with the ABTS results, although there was no significant correlation between TBARS and ABTS.

By comparing the results of the three indicators with the percentage of usable blastocysts, albeit non-significant, there was a moderate correlation between the percentage of usable blastocysts with ABTS (positive) and TBARS (negative). This result is consistent with the concept that oocytes exposed to oxidative stress are of lower quality.

Finally, our results suggest that antioxidant and pro-oxidant indicators may be used to evaluate oocyte quality; however, more studies on oxidative stress indicators are required, at the level of biomolecules and enzymatic systems, as well as their relationship with the entire oocyte maturation process. This study is preliminary; therefore, the findings described here will benefit from the evaluation of a larger number of samples. In addition, further studies are needed in order to elucidate the impact of the results presented here and the clinical outcomes (i.e. pregnancy or live birth rates).

## CONCLUSION

Our data demonstrate that patients of advanced-maternal-age display ovarian oxidative imbalance; however, in order to determine its effects on oocyte quality, we need more studies on larger sets of patients.
